# Effectiveness of non-pharmacological methods in reducing pain in pediatric patients and the role of nursing. Systematic review

**DOI:** 10.3389/fped.2025.1729847

**Published:** 2026-01-02

**Authors:** Juan Manuel Pérez-Pozuelo, Sonsoles Hernández-Iglesias, Mónica Raquel Pereira-Afonso, Abel Checa-Peñalver, Inmaculada García-Valdivieso, Ángel López-González, Sagrario Gómez-Cantarino

**Affiliations:** 1 Faculty of Physiotherapy and Nursing, University of Castilla-La Mancha, Toledo, Spain; 2 Health Sciences Faculty, Department of Nursery, University of Francisco de Vitoria Madrid, Pozuelo de Alarcón, Spain; 3 Faculty of Nursing, University of Castilla-La Mancha, Albacete, Spain

**Keywords:** non pharmacological, nursing, pain management, pediatrics, systematic review

## Abstract

**Introduction:**

The prevalence of pediatric pain has increased and is a leading cause of hospital attendance. Non-pharmacological treatments for pain relief in pediatrics are experiencing a boom.

**Methodology:**

Systematic review, following the quality guidelines of the PRISMA 2020 declaration. Previously determined inclusion and exclusion criteria were followed for the selection of studies.

**Results:**

The use of the vibrating and cold bee combined with a distractor is the most useful method, with virtual reality being the ideal distractor for the palliation of pediatric pain. The role of the nurse is essential to detect pain, choose the analgesia method and administer it.

**Discussion:**

The non-pharmacological techniques proposed and studied according to the selection of articles are useful in the reduction of pediatric pain according to the scales used in each study.

**Systematic Review Registration:**

https://www.crd.york.ac.uk/PROSPERO/view/CRD42025640378, identifier CRD42025640378.

## 1 Introduction

Pain has been the subject of study by healthcare professionals and researchers throughout history. As a result, the concept of pain has evolved and been interpreted from a broader perspective since its initial definition (1–3).

The International Association for the Study of Pain (IASP) defined pain in 1979 as “an unpleasant sensory and emotional experience associated with actual or potential tissue damage or described in terms of such damage” (4, 5).

In 2020, the IASP revised this definition, stating that “pain is an unpleasant sensory and emotional experience associated with, or similar to that associated with, actual or potential tissue damage.” Several aspects were considered in formulating this new definition: (1) pain is personal and can be influenced by various factors such as social, biological, and psychological aspects; (2) pain and nociception are distinct terms; (3) pain cannot be uniquely inferred from the activity of sensory neurons; (4) a person learns the term “pain” based on their life experiences; (5) when a person expresses a painful experience, it should be acknowledged and respected; (6) pain can have an adaptive function, but it can also affect a person's functioning and well-being (3, 6, 7).

A study conducted by the Analgesia and Sedation Working Group of the Spanish Society of Pediatrics in 2018 shows that the prevalence of pain in pediatric patients attending pediatric emergency departments is approximately 53%. Of this percentage, 25% report mild pain, 37% report moderate pain, and 38% report severe pain (8, 9). From this data, we can infer that pediatric pain is an issue that concerns all healthcare professionals, as it is often underestimated in this patient group (10).

There are numerous options for addressing pain, but non-pharmacological methods for pain relief in pediatrics have seen a significant rise in research and clinical practice. These methods are based on the understanding that anxiety, fear, uncertainty, and prior painful experiences can increase pain in pediatric patients and that these can be reduced or alleviated with techniques that do not rely on pharmacological interventions as the first line of treatment. They can be first line for clinical procedures, and for pain related to diseases, they play a complementary role. Due to their high effectiveness and ease of application, these methods are recommended in most cases where fear, pain, and anxiety are detected in pediatric patients (11–13).

All these techniques or methods can be applied by nursing staff, making nurses responsible for administering this analgesia and pain relief, with all the implications for the patient and the family. Ultimately, this is a nursing task that can be performed with adequate training and skill in non-pharmacological methods to achieve the same goal: pain reduction in pediatric patients (12–14).

## 2 Objectives

The overall objective of this systematic review is to evaluate the effectiveness of non-pharmacological interventions applied by the nursing team in reducing pain in pediatric patients and to analyze the role of nursing in the implementation of these strategies. From this overall objective, a specific objective arises: to analyze scientific evidence on the impact of non-pharmacological interventions applied by the nursing team in reducing pain in the pediatric population.

## 3 Method

### 3.1 Design of the research

A systematic review has been conducted, following the quality guidelines of the PRISMA 2020 statement (15). The Prospero repository was searched, and no similar studies were found; therefore, the following PICO (Patient, Intervention, Comparison, and Outcome) question was formulated: Are non-pharmacological methods effective in reducing pain in pediatric patients? The official citation provided by PROSPERO is as follows: Effectiveness of non-pharmacological methods in reducing pain in pediatric patients and the role of nursing. Systematic review. PROSPERO 2024. Available from https://www.crd.york.ac.uk/PROSPERO/view/CRD42025640378.

### 3.2 Keywords and thesaurus

For the bibliographic search, the MeSH (Medical Subject Headings) (16) and DeCS (Descriptors in Health Sciences) (17) thesaurus were used, based on the keywords in natural language shown in Table 1.

**Table 1:** Search strategy, descriptors and keywords used in this systematic review.

Databases (results)	Descriptors	Search strategy	Filters
PubMed	MeSH controlled descriptors: “Pediatrics”; “Pain”; “Pain management”; “Adult”.	((((“Pediatrics”[Mesh]) AND (“Pain”[Mesh])) OR (“Pain Management/methods”[Mesh] OR “Pain Management/nursing”[Mesh])) AND (Non-pharmacological)) NOT (“Adult”[Mesh] OR “Adults”)	Results by year: 2018–2024.
(39 articles)	Keyword: “Non-pharmacological”		Article type:
			Randomized clinical trial and Clinical Trial.
Cochrane	MeSH controlled descriptors: “Pediatrics”; “Pain”; “Pain management”.	“pediatric” in Title Abstract Keyword AND “pain” OR “Pain Management methods” OR “Pain Management nursing” in Title Abstract Keyword AND “non-pharmacological” in Title Abstract Keyword NOT “adult” OR “adults” in Title Abstract Keyword	Customized timeframe: 2018–2024.
(157 articles)	Keyword: “Non-pharmacological”		
ScienceDirect	Controlled descriptors from the database: “Pediatrics”; “Pain”; “Pain management”; “Adult”.	Pediatrics AND (Pain OR “Pain Management methods” OR “Pain Management nursing”) AND (Non-pharmacological) AND NOT (Adult OR Adults)	Selection of years: 2018–2024.
(181 articles)	Keyword: “Non-pharmacological”		Article type: Research article.
			Open access and open archive
SciELO	Controlled descriptors from DeCS: “Pediatrics”; “Pain”; “Pain management”; “Adult”.	(Pediatrics OR Pediatria OR Pediatría) AND (Pain OR Dor OR Dolor OR “Pain Management methods” OR “Métodos de manejo da dor” OR “Métodos de manejo del dolor” OR “Pain Management nursing” OR “Enfermagem no manejo da dor” OR “Enfermería en el manejo del dolor”) AND (“Non-pharmacological” OR “Não farmacológico” OR “No farmacológico”) AND NOT (Adult OR Adults OR Adulto OR Adultos)	Year of publication: 2018–2024.
(14 articles)	Keyword: “Non-pharmacological”		

*Source*: Own elaboration.

### 3.3 Bibliographic search procedure

#### 3.3.1 Search strategy

Considering the specific syntax and distinct filters of each database, we present in Table 1 the adapted search strategies and the filters applied. For the selection of articles, it was considered that (1) the title or abstract should contain the descriptors; (2) the type of study was considered; and (3) the year of publication should be between 2018 and 2024.

#### 3.3.2 Selection criteria

Next, we present the inclusion and exclusion criteria used in the bibliographic search. This allowed us to refine the search and ensure alignment with the established objectives.

The inclusion criteria are: (1) Language: Spanish, English, and Portuguese; (2) studies published between 2018 and 2024; (3) studies that compare the use of non-pharmacological approaches applied by the nursing team in the management of pediatric pain; and (4) randomized clinical trial studies.

The exclusion criteria are: (1) studies with a low level of evidence; (2) studies with a high risk of bias; (3) studies that do not present relevant data or duplication of information and irrelevance to the research topic.

#### 3.3.3 Bibliographic search

Following the quality criteria of the PRISMA 2020 statement, the selection of studies was carried out in several phases.

An initial search was conducted on PubMed to identify potential articles for the study and to test the descriptors, but without the use of filters at this stage. After the researchers made the necessary adjustments and developed the search strategy, as described in Table 1. Therefore, search filters were applied, resulting in a systematic search in 4 different databases (PubMed, SciELO, Science Direct, and Cochrane). The search took place from April 2nd to April 9th, 2025, and all search results were exported according to the availability of each database.

After exporting the databases, software (Zotero) was used to analyze and remove duplicate studies. Subsequently, the titles and abstracts of the articles were examined independently by two researchers (J.M.P.-P.; S.H.-L.), selecting and excluding articles according to the inclusion and exclusion criteria. Afterwards, the articles included were discussed by these researchers, and a third researcher (S.G.-C.) was called in to evaluate the articles that presented discrepancies in inclusion/exclusion.

**Figure 1:**
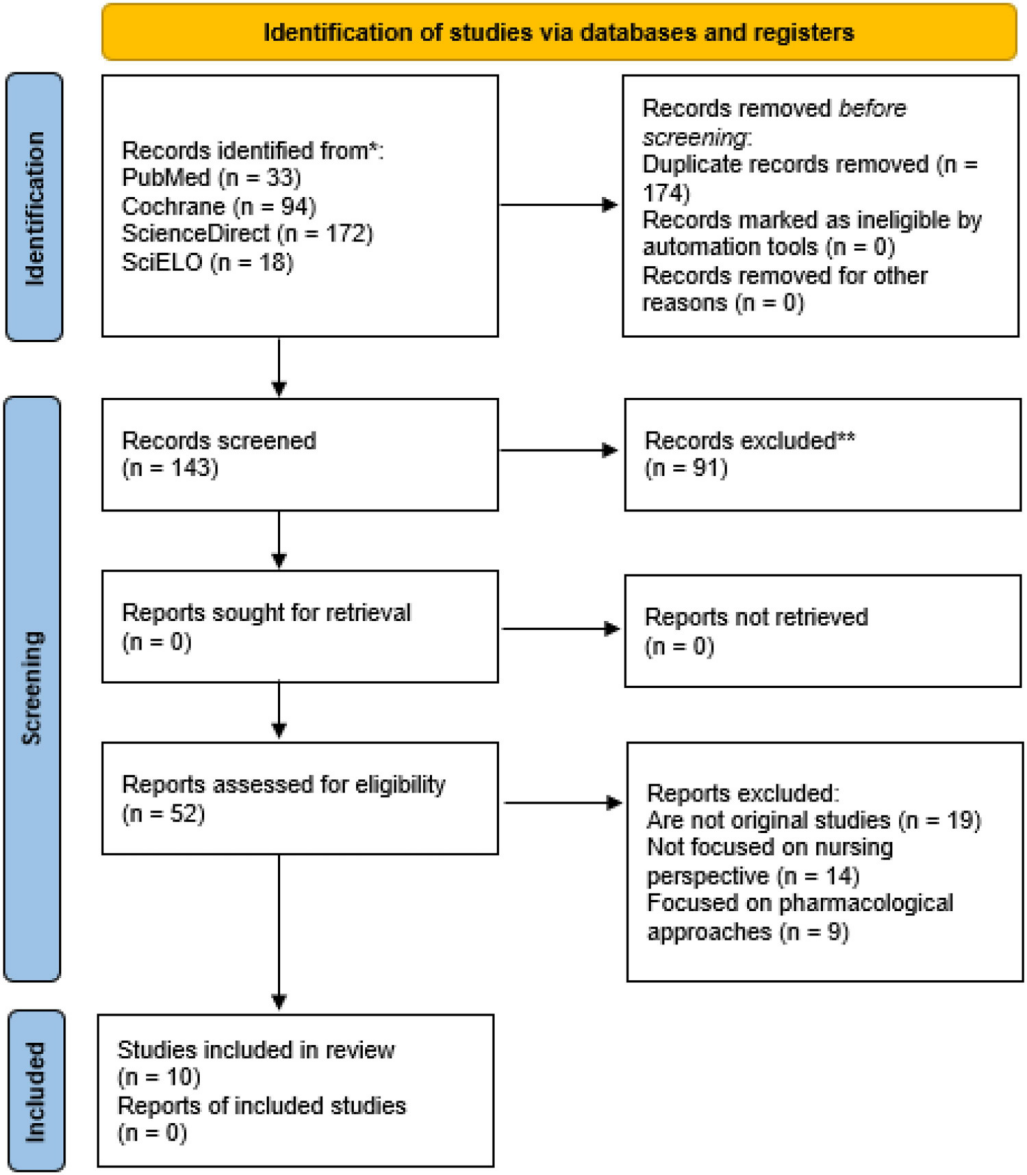
PRISMA flow diagram of the study search process. Source: Own creation based on the PRISMA flow diagram (15).

Finally, after the exclusion process, the articles were read in full to see if they fully met the inclusion criteria. Of these, original studies that were not randomized clinical trials, studies that did not contemplate the application of non-pharmacological techniques by the nursing team, and studies that did not sufficiently answer the guiding question were excluded. At the end of this selection, the articles that make up the sample of this systematic review were included. The complete flowchart of the study can be seen in Figure 1.

### 3.4 Assessing the quality and level of evidence

To assess the methodological quality of the included articles, the Mixed Methods Appraisal Tool (MMAT) was used (18). The choice is justified by its proven validity and reliability in the concurrent assessment of different study designs, allowing for a standardized and efficient analysis.

Although the review has randomized clinical trials as an inclusion criterion, the MMAT was used specifically and exclusively for category 2 (quantitative randomized controlled trials), which refers entirely to this type of study. This category assesses crucial criteria for the internal validity of this design, including: (1) the adequacy of randomization, (2) the comparability of groups at baseline, (3) the integrity of outcome data, (4) blinding of assessors, and (5) adherence to the designated intervention, as described in Table 2.

**Table 2:** Methodological quality assessment using the mixed methods appraisal tool (MMAT).

Articles	2.1	2.2	2.3	2.4	2.5	Sensitivity analysis (best evidence and quality)
Ardahan Akgül et al. (19)	YES	YES	YES	YES	YES	The study is well executed, with no loss to follow-up and a triple-blind design. The sample size was based on power calculations, and the *p*-values are highly significant, indicating precision and a low risk of methodological bias. The main limitation was the application of the intervention on a single day, which limits the external validity for long-term treatments but does not invalidate the quality of the trial.
Gil Piquer et al. (27)	YES	YES	YES	NO	YES	The study features robust randomisation, with no loss of data, and the groups are homogeneous at baseline. However, it lacks blinding, which compromises internal validity in terms of the risk of bias in the assessment of subjective outcomes evaluated by nurses and patients who were aware of the intervention.
Kjeldgaard Pedersen et al. (28)	YES	YES	YES	NO	YES	The study presents solid methodological quality, considering the randomised crossover design with 24 sequences, which effectively controls for individual confounding variables and order effects. The use of multiple control groups reinforces internal validity by isolating the immersion effect. However, it has one limitation: the lack of blinding of the evaluators, which introduces a risk of bias in the measurement of the Pressure Pain Threshold, as the evaluator knew the intervention the child was receiving. Nevertheless, it presents narrow confidence intervals and the effect is large and consistent.
Yaz et al. (24)	YES	YES	YES	NO	YES	The study demonstrates methodological quality, random sequence generation, allocation concealment, and guaranteed baseline comparability. The main limitation is the absence of blinding of outcome assessors, which introduces a risk of detection bias, as assessors may overestimate the effect of the intervention due to expectation of benefit. However, the effect size is large and statistically significant, supporting the moderate rating.
Gotkas; Avci (22)	YES	YES	YES	NO	YES	The study presents adequate methodological quality in terms of randomisation and data integrity. The sample size was based on power calculation. The main limitation was the absence of blinding of the outcome assessor, and given that the outcomes are subjective and self-reported under the supervision of the investigator, there is a moderate risk of detection bias. However, the magnitude of the observed effect suggests that the results are reliable.
Yildizeli Topcu et al. (25)	YES	YES	YES	NO	YES	The study has strengths such as computerised randomisation and excellent data integrity with minimal losses. However, internal validity is compromised by the lack of blinding of assessors, which may influence observation, self-reports and prior knowledge of allocation, which could have influenced nurses' scores and possibly children's behaviour. Nevertheless, the study is reliable in terms of its technical execution, but its results should be interpreted with caution due to the risk of measurement bias.
Bergomi et al. (20)	YES	YES	YES	NO	YES	The evidence from this study was considered moderate due to the risk of bias arising from the lack of blinding of assessors and participants, which could lead to subjective outcomes. The sample size was adequately calculated to ensure statistical power, avoiding degradation due to imprecision. Rigorous randomisation supports internal validity within the limits allowed by this design.
Dur et al. (26)	YES	YES	YES	NO	YES	The study has moderate to high methodological quality, with strengths such as computerised randomisation, baseline comparability between groups, and complete data integrity. However, it was not blinded to the evaluators. Since pain was assessed using an observational scale (NIPS) by the professionals who administered or witnessed the intervention, there is a considerable risk of detection bias, which could overestimate the magnitude of the effect on subjective outcomes; however, physiological data are less susceptible to this bias.
Fitri et al. (21)	YES	YES	YES	NO	YES	The magnitude of the effect is high and the confidence intervals inferred from the standard deviation are accurate. However, the study presents a risk of detection bias due to the lack of blinding of the evaluators, which could influence the assessment of the perceived effectiveness of the intervention on the behavioural scales, resulting in moderate evidence.
Metreş; Yildiz (23)	YES	YES	YES	NO	YES	The study is characterised by computerised randomisation, comparability and well-defined data integrity. One limitation is the lack of blinding of assessors in the behavioural observation and intervention phases, resulting in a risk of detection bias. However, physiological outcomes are objective and less susceptible to this bias.

2.1—Is randomization appropriately performed?.

2.2—Are the groups comparable at baseline?.

2.3—Are there complete outcome data?.

2.4—Are outcome advisors blinded to the intervention provided?.

2.5—Did the participants adhere to the assigned intervention?.

*Source:* Own creation based on MMAT Tool (18).

The application of the MMAT allowed us to identify the level of methodological rigor of the nursing interventions analyzed, ensuring that the evidence on non-pharmacological practices for the management of pediatric pain applied by the nursing team was based on studies with verified methodological quality.

## 4 Results

### 4.1 Characteristics of the studies

In this review it has been selected 10 studies that meet the inclusion and exclusion criteria. Regarding the design of the included studies, it has been found that 7 of the 10 articles are randomised controlled trials (19–26). An article is a multicenter randomized clinical trial (27). And the last article is a randomised crossover trial (28).

All the articles that have been selected in this systematic review study whether non-pharmacological analgesia methods are effective in reducing pain in children. The sample size of all these studies ranged from *n* = 64 to *n* = 150. The age of the samples ranged from newborns (NB) to children aged 14 years, thus covering almost the entire pediatric age range. This research has been carried out in different health areas such as hospital emergency departments (22), hospitalised children (19, 21, 25, 27), children seen in primary care or outpatient clinics (20, 24, 28), children admitted to the Neonatal Intensive Care Unit (NICU) (23, 26), among others.

These samples have come from different countries such as Turkey, where 6 articles have been selected (19, 22–26); Spain, where 1 article has been selected (27); Denmark, where 1 article has been selected (28); Italy, where 1 article has been selected (20) and Indonesia, where 1 article has been selected (21).

All investigations have studied a specific non-pharmacological method. Thus, out of the 10 studies 1 of them studied lavender inhalation (19), 2 the use of virtual reality (27, 28), 2 the use of vibration and cold (20, 24), 1 the use of auditory and visual distraction techniques (22), 1 the use of breastfeeding (21), 1 the use of chewing gum (25) and 1 the application of Yakson and Gentle Human Touch (26), 1 the use of the ROP position (23).

Table 3 below shows the main results and characteristics of the 10 selected studies.

**Table 3:** Characteristic of the included studies.

Author, year and country	Title	Study design	Objetive	Characteristic of the sample	Results	Limits
Ardahan Akgül et al.. Türkiye (19)	Effectiveness of lavender inhalation aromatherapy on pain level and vital signs in children with burns: a randomized controlled trial	Randomised controlled trials	To assess the effectiveness of aromatherapy through inhalation of lavender oil applied before dressing changes on vital signs and pain levels in children with burns.	*N* = 108	Inhalation aromatherapy applied before dressing in children with burns decreases and reduces pain levels and stabilizes vital signs.	Lavender aromatherapy was applied only for one day.
				Age: 2 months—7 years		
				Dr. Behçet Uz Children's Diseases and Surgery Hospital		
Gil Piquer et al. Spain (27)	Usefulness of virtual reality in the management of pain associated with venepuncture: a multicentre randomized clinical trial	Multicentre randomised clinical trials	To evaluate the efficacy of virtual reality (VR) in reducing pain and anxiety associated with scheduled blood draws.	*N* = 83	Pain associated with venipuncture was reduced, supporting the use of virtual reality as a distractor for pediatric pain reduction.	The inability to blind the study due to the same nurse being involved for all participants.
				Age: 7–12 years		
				Two regional hospitals located in the same healthcare area of the Valencian Community, Spain		
Kjeldgaard Pedersen et al. Denmark (28)	Virtual reality increases pressure pain threshold and lowers anxiety in children compared with control and non-immersive control-A randomized, crossover trial	Randomised crossover trials	To assess the effect of VR on pain pressure threshold (PPT) and anxiety levels measured with the Modified Yale Preoperative Anxiety Scale (mYPAS) in children in a controlled experimental setting.	*N* = 72	Virtual reality was beneficial in improving pain pressure threshold (PPT) and anxiety compared to control interventions. It can be concluded that immersive virtual reality has a modulatory effect on pain and anxiety.	The sample size is small.
				Age: 6–14 years		
				Outpatient Orthopedic Clinic of Aarhus University Hospital		
Yaz et al. Türkiye (24)	The effect of vibrating cold application and puppet use on pain and fear during phlebotomy in children: A randomized controlled study	Randomised controlled trials	This study was conducted to assess the effects of bee buzzing and puppet use on pain and fear during phlebotomy in children.	*N* = 105	The use of the Buzzy bee is effective in reducing pain, and its combination with puppets is effective in decreasing the fear experienced by children aged 3 to 6 years during phlebotomy.	Pain intensity and fear levels were evaluated only by the parents and the nurse. The double-blind technique could not be used.
				Age: 3–6 years		
				Pediatric Phlebotomy Service of a University Hospital		
Gotkas; Avci. Türkiye (22)	The effect of visual and/or auditory distraction techniques on children's pain, anxiety and medical fear in invasive procedures: A randomized controlled tria	Randomised controlled trials	To determine the effects of visual and/or auditory distraction techniques applied to children aged 7 to 12 years during invasive procedures on pain, anxiety, and medical fear.	*N* = 144	Pain, anxiety, and medical fear levels after invasive procedures were lower in the intervention groups compared to the control group.	Only children admitted to the emergency department were studied. Only the children's statements were considered.
				Age: 7–12 years	No differences were found between the three different distraction techniques used, but virtual reality was more effective in reducing anxiety levels.	
				Pediatric Emergency Service of a public hospital		
Yildizeli Topcu et al. Türkiye (25)	Effect of Gum Chewing on Pain and Anxiety in Turkish Children During Intravenous Cannulation: A Randomized Controlled Study	Randomised controlled trials	To investigate the effect of chewing gum on pain and anxiety during intravenous (IV) cannulation in children.	*N* = 73	Reports from the children and the nurse showed that the pain level in the experimental group was significantly lower than in the control group.	The study sample is small.
				Age: 6–12 years		
				General Pediatric Unit of a University Hospital in the Trachy region, Turkey		
Bergomi et al. Italy (20)	Efficacy of Non-pharmacological Methods of Pain Management in Children Undergoing Venipuncture in a Pediatric Outpatient Clinic: A Randomized Controlled Trial of Audiovisual Distraction and External Cold and Vibration	Randomised controlled trials	To evaluate two non-pharmacological techniques, vibration combined with topical analgesia using the Buzzy® device, and cartoons, in terms of pain relief and anxiety during venipuncture in children.	*N* = 150	Effectiveness of non-pharmacological pain management methods during venipuncture.	The study was not double-blinded, and it did not assess nurse satisfaction regarding pain management.
				Age: 5–12 years	Distraction with cartoons was more effective in reducing pain perception compared to Buzzy and the combination of Buzzy and cartoons.	
				Outpatient Pediatric Clinic of San Matteo Hospital, Pavia	Anxiety levels in both children and parents were lower with non-pharmacological methods. This way, we involve nurses in pediatric care.	
Dur et al. Türkiye (26)	The effect of Yakson and Gentle Human Touch methods on pain and physiological parameters in preterm infants during heel lancing	Randomised controlled trials	To determine the effects of the Yakson and Gentle Human Touch methods on pain and physiological parameters (heart rate and oxygen saturation) during heel prick procedures in premature newborns.	*N* = 90	The Yakson and Gentle Human Touch groups showed significantly lower pain scores during and after heel prick compared to the control group (*p* < .001).	Researchers could not be blinded during group assignment. This limitation could be addressed by ensuring that participants and researchers involved in other aspects of the trial are blinded.
				Age: Birth—32nd and 36th week of gestation (average gestational age of 33.44 ± 1.74 weeks)		
				Neonatal Intensive Care Unit of a hospital in Türkiye		
Fitri et al. Indonesia (21)	Modified Sensory Stimulation Using Breastmilk for Reducing Pain Intensity in Neonates in Indonesia: A Randomized Controlled Trial	Randomised controlled trials	To compare the analgesic effects of Sensory Saturation with sucrose (SSS), SSS with breast milk (SSB), and oral sucrose alone (S24%) in neonates undergoing venipuncture.	*N* = 108	SSB and SSS were more effective than S24% (*p* = 0.001). No differences were observed between SSB and SSS (*p* = 0.669).	The study was conducted only at the time of needle insertion. Video recording was not used during the study observations, and the distinction between neonates previously exposed to painful procedures and those who had not was not made.
				Age: Birth—32 WG and up to 48 h of life		
				Neonatal Wards of two provincial government hospitals in West Java, Indonesia		
Metreş; Yildiz Türkiye (23)	Pain Management with ROP Position in Turkish Preterm Infants During Eye Examinations: A Randomized Controlled Trial	Randomised controlled trials	To assess the effects of baby positioning on pain, heart rate, oxygen saturation, and crying time during the ocular examination.	*N* = 70	Preterm neonates in the experimental group had lower pain scores than those in the control group. Heart rate was higher in the control group compared to preterm neonates in the experimental group. Oxygen saturation was not significant between groups before, during, or after screening. Crying duration was shorter in the experimental group compared to the control group.	The effects of the inverted kangaroo care position on pain were not assessed using an objective measurement tool.
				Mean Gestational Age: 29.78 ± 2.07 weeks in the experimental group and 29.57 ± 1.89 weeks in the control group		
				Neonatal Intensive Care Unit of the Istanbul Maternal-Infant Training and Research Hospital		

*Source*: Own creation based on the studies included in the systematic review.

### 4.2 Development of the results

#### 4.2.1 Lavender inhalation

In research conducted in Turkey by Ardahan et al., which studied the effect of lavender inhalation on pain reduction in the bandage after burn healing, it was found that in the measurements with the FLACC (The Face, Legs, Activity, Cry, Consolability Scale) Pain Scale (29) after bandaging (after 1 min and 30 min), the measured pain levels were lower in the Lavender-15 (application of lavender 15 min before dressing) and Lavender-60 (application of lavender) groups (application of lavender 15 min before dressing).

In post-dressing measurements (both at 1 min and 30 min), pain levels were lower in the Lavender-15 and Lavender-60 groups compared to the control group (*p* = 0.000). After applying Bonferroni correlation analysis, there is a difference between the lavender- and control groups with respect to pain levels measured at 1 min (*p* = 0.000) and 30 min (*p* = 0.000) after bandaging. There is also a difference between the lavender-60 and control groups with respect to pain levels measured at one minute (*p* = 0.000) and 30 min after bandaging in burn healing (19).

#### 4.2.2 Vibration and cold

In a study conducted in Turkey by Yaz et al., with the aim of evaluating the effects of bee buzzing (vibration and cold) and the use of puppets on pain and fear during phlebotomy in children, results were obtained with lower pain scores according to the Wong-Baker Faces Pain Rating Scale (30) in the bee buzzing group than in the puppet and control groups. A statistically significant difference was observed between these groups, with the bee group having lower fear scores than the other groups (*p* < 0.05). A statistical difference was evident between groups based on parent-reported pain scores (*p* < 0.001). Statistical difference was found between groups according to nurse-reported pain scores (*p* < 0.001). Statistical difference was found between the Bee Buzzy or Puppet group and the Control group according to parent- and nurse-reported pain scores (*p* < 0.05) (24).

Compared to another study by Bergomi et al. that gives similar results, which also studied the application of cold and vibration with the aim of evaluating the use of vibration combined with topical cryotherapy analgesia using the Buzzy® device and cartoons (in terms of pain and anxiety relief during venipuncture in children), results were obtained in which children's perceived pain according to the Wong Baker Faces Pain Scale (WBFP) (30) had lower scores in the non-pharmacological intervention groups compared to the non-intervention group. The difference was statistically significant in the cartoon group (*p* = 0.02). However, Buzzy® was highly effective in children under 9 years of age (*p* = 0.04). Significant efficacy was obtained in the Buzzy® and cartoon group (*p* = 0.04) for the nurse's perception of the child's pain, and in the Buzzy® group for the mother's perception of the child's pain (*p* = 0.002) (20).

#### 4.2.3 Visual and auditory distraction techniques

In a study conducted in Turkey by Gotkas & Avci using visual and auditory distraction techniques such as the use of a kaleidoscope, music and virtual reality, the results show that the level of pain measured in the control group patients after an invasive procedure was higher on the Wong-Baker Faces Pain Rating Scale (30) than the pain levels of the children who had these distraction techniques used (*p* < 0.05).

The level of anxiety experienced by these children after a painful technique was higher in the control group than in the intervention group (*p* < 0.001). If we compare the 3 distraction techniques, the most effective in the study was virtual reality, as anxiety levels were lower in this group than in the music and kaleidoscope groups (22).

Several studies have also been included that study the use of Virtual Reality (VR) in pain palliation in pediatrics, specifically in the study carried out in Spain by Gil et al. (27), results of milder pain were obtained in the VR group (with a statistically significant difference). In the VR group, about 83% of the children had no or mild pain, compared to about 57% in the control group (*p* = 0.012). Thus, there was a 26% reduction in terms of pain frequency in children (27).

Another study conducted in Denmark by Kjeldgaard et al. measured whether immersive VR alleviates pain and anxiety in children by applying a hand-held algometer that applies a certain pressure, concludes similarly. Children who received immersive VR significantly increased their pain threshold compared to the control group (*p* < 0.0001). The same was true for the anxiety levels they measured, which decreased more markedly in the immersive VR group than in the non-immersive VR groups (*p* < 0.0001) (28).

#### 4.2.4 Breastfeeding

Another non-pharmacological method used is breastfeeding and in the study by Fitri et al. conducted in Indonesia comparing the analgesic effects of Sensory Saturation (SS) with sucrose (SSS), SS with breast milk (SSB) and oral sucrose alone (S24%) in neonates undergoing phlebotomy, yielded the following results: mean pain intensity in neonates according to the Premature Infant Pain Profile-Revised (PIPP-R) (31) scale who received SSS was 2.222 ± 1.098 and in neonates who received BSS was 2.028 ± 1.055. The mean pain intensity in neonates receiving S24% alone was 5.222 ± 1.290. The mean pain intensity in neonates receiving BSS was the lowest and the mean in those receiving S24% was the highest. Therefore, the analysis shows that the pain intensity in the BSS intervention group was the lowest and significantly unequal to that of the S24% intervention group (*p* = 0.001), but not significantly different from that of the SSS intervention group (*p* = 0.669) (21).

#### 4.2.5 Chewing gum

A study conducted in Turkey by Yildizeli et al. investigated the effect of chewing gum and pain reduction during intravenous cannulation using the Children's Anxiety and Pain Scale-CAPS (32). Results showed that children who chewed gum during the pain technique experienced less pain than children in the control group. These pain ratings by the nurses and the children themselves reinforce that chewing gum could decrease the pain experienced during a procedure and/or technique considered painful (*p* = 0.044). The children's pain levels were significantly lower for the experimental group (mean = 1.27 ± 0.96) than for the control group (mean = 1.42 ± 0.91, *p* = 0.040). The nurse assessed pain levels were significantly lower for the experimental group (mean = 0.89 ± 1.04) than for the control group (mean = 1.25 ± 0.91, *p* = 0.044) (25).

#### 4.2.6 Method Yakson and Gentle Human Touch

The study results demonstrated that both the Yakson method and Gentle Human Touch were significantly effective in reducing pain and stabilizing heart rate in premature infants during heel prick. Specifically, babies in the Yakson and Gentle Human Touch groups showed significantly lower pain scores on the Neonatal Infant Pain Scale than the control group during and after the procedure, with statistically significant differences (*p* < .001). Similarly, heart rate remained lower and more stable in the groups that received touch intervention, while the control group exhibited higher rates indicative of stress (*p* < .001). On the other hand, no statistically significant difference was found in oxygen saturation levels between the three groups (*p* > .05). According to the authors, this occurred because all babies, including those in the control group, received a pacifier, which in itself may have helped maintain oxygenation (26).

#### 4.2.7 ROP position

A study conducted in Turkey by Metres & Yildiz assessed the effects of infant position on pain, heart rate, oxygen saturation and crying time during an eye examination. Results showed that preterm NBs in the experimental group had lower pain scores according to the PIPP-R scale (31) at baseline, at the end and after screening than those in the control group.

In a paired comparison between the groups, there was a 4.17 ± 1.95 point increase in the pain score of preterm infants in the experimental group at the start of screening (*p* = 0.001), a 3.00 ± 2.03 point increase at the end of screening (*p* = 0.001) and a 1.09 ± 1.22 point increase after screening (*p* = 0.001) compared to the mean score before screening (*p* = 0.001). When compared with the mean scores at baseline, the decrease of 1.17 ± 2.15 points (*p* = 0.004) in the mean pain score at the end of the examination and the decrease of 3.09 ± 2.08 points (*p* = 0.001) after the examination (*p* < 0.01) were found to be significant. When compared to the values at the end of the examination, the decrease of 1.91 ± 1.70 points (*p* = 0.001) in the mean pain score after the examination (*p* < 0.01) was also significant (23).

#### 4.2.8 The role of nursing in pediatric pain reduction using non-pharmacological analgesia

In the study investigating the use of lavender oil to reduce pain in the pediatric burn population, the result is that the nurse can reduce pain in children who have had a burn by simply applying lavender oil. Thus, we use an easy and feasible method that has been found in this study to reduce pain (19).

In another study looking at cold, vibration and cartoons. The perception of nursing on the child's pain in the bee and cartoon group has a significant efficacy. The difference was statistically significant in the bee and bee and cartoon groups for the nurses' perception of the child's anxiety (20).

Regarding the SS study we also obtain results that involve nursing as according to the study the nursing profession is the one in charge of carrying out the education to perform BSS, as it is a cheap, easy method and that benefits are obtained in the reduction of child pain. The nurses in this study are in charge of all data collection, pain assessment, venipuncture, among other things (21).

All previous studies demonstrate that the effectiveness of these interventions largely depends on their suitability to the child's developmental stage and the specific characteristics of the procedure. Similarly, the transversal role of nursing is highlighted, since the active involvement of nursing professionals in the application, supervision, and evaluation of these interventions is a fundamental element for their correct implementation and to ensure a comprehensive approach to pediatric pain management, as shown in Table 4.

**Table 4:** Analytical comparison of non-pharmacological interventions.

Intervention category	Pain scales used	Main effects (effect magnitude)	Similarities	Differences	Analytical interpretation
Aromatherapy (Lavender) (19)	FLACC—Face, Legs, Activity, Cry, Consolability	Significant reduction in pain at 1 and 30 min post-dressing in Lavender-15 and Lavender-60 groups (*p* = 0.000).	Simple, quick intervention with good tolerance.	Similar analgesic effect between both lavender timings.	Suitable for repeated dressing procedures; consistent analgesic effect.
Vibration + cold (20, 24)	Wong-Baker Faces Pain Rating Scale	Lower pain and fear in child/parent/nurse reports (*p* < 0.05; *p* < 0.001). Greater efficacy in younger children (*p* = 0.04).	Both combine distraction and peripheral sensory input.	Bee Buzzy® more effective in <9 years.	Strongest analgesic effect for venipuncture; multimodal stimulation superior to single techniques.
Visual/auditory distraction and Virtual Reality (22, 27, 28)	Wong-Baker Faces Pain Rating Scale; pressure algometry	Lower pain and anxiety (*p* < 0.001). 83% of VR group with no/mild pain vs. 57% control (*p* = 0.012). Higher pain threshold and reduced anxiety with immersive VR (*p* < 0.0001).	VR consistently shows strongest effect.	Immersive VR > music/kaleidoscope.	VR provides the most potent analgesic and anxiolytic effect due to cognitive immersion.
Sensory stimulation + breastfeeding (21)	Premature Infant Pain Profile-Revised	Lowest pain scores in BSS; significant difference vs. S24% (*p* = 0.001).	Highly effective in neonates.	SSS and BSS similar; S24% inferior.	Optimal neonatal analgesia; breastfeeding adds affective benefit.
Chewing gum (25)	Children's Anxiety and Pain Scale	Significantly lower pain in children (*p* = 0.040) and nurses' assessment (*p* = 0.044).	Simple and inexpensive.	Less effective than VR or vibration + cold.	Suitable for moderate-pain procedures; good feasibility.
Yakson and Gentle Human Touch (26)	Neonatal Infant Pain Scale	The Yakson and GHT groups showed significantly lower pain scores during and after heel prick compared to the control group (*p* < .001).	Both techniques involve tactile stimulation.	Yakson's method involves more circular movements, while the Gentle Human Touch method is more static.	The results suggest that both Yakson and Gentle Human Touch are highly effective non-pharmacological strategies for mitigating acute pain and physiological stress in premature infants.
ROP positioning (23)	Premature Infant Pain Profile-Revised	Significant pain reduction at all times (*p* ≤ 0.001); decreased crying and physiological instability.	Effective for ophthalmologic procedures.	Limited to preterm infants.	Positioning as a safe, effective method for procedural neonatal pain.
Role of nursing (19–21)	Corresponding pain scales	Nurses responsible for administering, monitoring and evaluating all interventions.	Nursing involvement is central in all studies.	Variability according to clinical context.	Successful implementation depends strongly on nursing leadership and training.

Source: own elaboration.

## 5 Discussion

The aim of this systematic review is to identify the effectiveness of non-pharmacological methods in the reduction of pain in pediatric patients in hospital emergency departments and the role of nursing. Ten articles were selectively selected, meeting the imposed inclusion criteria. The results obtained are related to the reduction of pediatric pain using a non-pharmacological approach and highlighting the role of the nursing profession in its administration and control.

Pain is a subjective sensation, difficult to measure, especially in the pediatric population or young children who cannot or insufficiently express themselves verbally. Pediatric pain is often under-diagnosed and under-treated, although it is quite common (33).

The use of non-pharmacological methods to reduce pain is being well received by the population, although there are still many myths to be debunked and studies to be carried out to confirm the effectiveness of these methods, which may include techniques such as breastfeeding, the use of 24% oral sucrose, the mother-kangaroo method, the use of the Buzzy bee, the application of local cold, the use of distractors (VR, cartoons, etc.), the use of aromatherapy, among others (19–22, 24, 27, 28).

Heterogeneity was observed in the measurement instruments (FLACC, WBFPRS, PIPP-R, and CAPS), which demands caution in the quantitative synthesis of the data. However, the consistency of positive outcomes across these different metrics suggests clinical robustness of the nursing interventions evaluated. The fact that non-pharmacological strategies demonstrated efficacy across various evaluative parameters reinforces the external validity of these practices in different social and clinical contexts of pediatric non-pharmacological care.

According to a study led by Eijlers et al. the use of VR as a distraction method is showing promise in distracting children from the anxiety and pain caused by various invasive techniques. He conducted a meta-analysis of 14 studies based on pain and 7 on anxiety, which showed the efficacy of VR in reducing both. These results are in line with the results obtained in the studies by Gil Piquer et al. and Kjeldgard et al. of the systematic review. However, according to the authors of the study by Eijlers et al. the sample of children was not large and, moreover, only the use of VR for burns in younger children was studied (34). Although these studies have a small sample size, the results showed statistical significance, and even though it is not possible to generalize their findings, they already point to evidence of non-pharmacological care provided by the nursing team (23, 27, 28).

In line with the above, another study conducted by Susam et al., which studied the use of the vibrating and cold bee to reduce pain in children in venipuncture, found that pain was significantly lower in the experimental group than in the control group, and that the experimental group had better pain control, as also expressed by Yaz et al. and Bergomi et al. in the studies selected for the systematic review. However, the study by Susam et al. has limitations such as the use of the bee in pediatric population with altered emotion state problems (35). The use of the bee in conjunction with other distraction techniques could help the nursing professional to alleviate the pain felt by the pediatric patient in invasives techniques.

In another study carried out by Oliveira et al., which investigates audiovisual distraction through short animated films for pain relief in pediatric patients, results were obtained with a significant difference between the periods with and without distraction in both groups of the study. Scores on pain scales were lower during the screening of these short films, compared to scores without short films (36). These results are in line with those obtained by Gotkas et al. in their study also included in the systematic review conducted. Therefore, audiovisual distraction is a good reducer of perceived pain intensity in the pediatric patient.

According to a study conducted by Lewis et al., which aimed to assess the use and overall knowledge of pharmacological interventions among clinical nurses, as well as the efficacy of pain treatment in a tertiary care medical centre. It can be concluded that this study has advanced the management and use of non-pharmacological methods by clinical nurses and identified opportunities for future studies and research as well as for the education of nursing students. Therefore, it should incorporate this type of initiative into their healthcare practice and be aware of the risk and consequences of the use of drugs, specifically opioids, in order to comprehensively address pain and, in this case, pediatric pain (14).

In line with these findings, the fundamental role of nursing is highlighted not only in administering the interventions, but also in assessing pain, educating families, and supporting clinical decision-making. This role becomes particularly relevant in contexts where the aim is to reduce the use of pharmacological analgesics, especially opioids, and to promote non-invasive, safe, and culturally adapted strategies.

The predominance of research in distinct contexts, ranging from Turkey and Indonesia to European systems (Spain, the United Kingdom, and Nordic countries), may reflect different degrees of autonomy of the nursing team and of healthcare models. The successful implementation of these interventions in these locations indicates that environments that value integrative practices and give nursing a leading role favor the adoption of non-pharmacological strategies. Therefore, the replicability of these results in other settings depends not only on the technique itself, but also on the structuring of work processes that allow for such nursing intervention.

### 5.1 Limitations

These results are promising and should encourage further study of non-pharmacological methods in the reduction of childhood pain in order to really know more about their efficacy and effectiveness in the application of nursing care. In order to do so, more studies with an adequate and large sample size should be carried out, as some of the limitations of this systematic review are the sample size of some of the selected articles. In addition to the duration of the effect of these non-pharmacological methods, as long-term effects are not studied, the scales used in the different studies are different, which may lead to biased reporting of variables such as pain. Also, the countries of the selected articles are very varied and the physiological and behavioural characteristics of the sample in each country may vary and lead to a sample that is too heterogeneous.

Another future line of research would be to study the impact of the role of nursing in leading pain detection and administering these alternative therapies and non-pharmacological methods to pharmacological analgesia (pre- and post-nursing intervention studies).

## 6 Conclusion

This systematic review indicates that non-pharmacological interventions can contribute meaningfully to reducing pain, anxiety, and fear in pediatric patients; however, the magnitude and consistency of these effects vary across studies. The evidence base is heterogeneous, with differences in study designs, sample sizes, clinical settings, and the pain assessment scales used, making direct comparison challenging and limiting the generalizability of findings. For this reason, no single intervention can be considered universally superior, and the effectiveness of each technique appears to depend on contextual factors such as the child's developmental stage, the nature of the procedure, and the clinical environment.

Despite this variability, non-pharmacological methods represent a valuable set of strategies that can enhance the quality of pediatric care and support a more holistic, humanized approach. Techniques such as distraction-based interventions, sensory stimulation, virtual reality, vibration and cold application, breastfeeding, and parental contact have all demonstrated benefits in specific contexts. Their incorporation into clinical practice should therefore be guided by an individualized assessment of patient needs, resource availability, and the procedural characteristics involved.

Nursing professionals play a pivotal role in the successful application of these interventions. Their expertise in pain assessment, patient and family education, and clinical decision-making is essential for determining the most appropriate non-pharmacological strategies and for ensuring their safe and effective implementation. Continued research is needed to strengthen the evidence base, promote methodological standardization, and explore how these interventions can be optimized and adapted across diverse healthcare settings.

## Data Availability

The original contributions presented in the study are included in the article/Supplementary Material, further inquiries can be directed to the corresponding author.
